# Resveratrol Oligomers, Plant-Produced Natural Products With Anti-virulence and Plant Immune-Priming Roles

**DOI:** 10.3389/fpls.2022.885625

**Published:** 2022-05-27

**Authors:** Ji Eun Kang, Nayeon Yoo, Byeong Jun Jeon, Beom Seok Kim, Eui-Hwan Chung

**Affiliations:** ^1^Institute of Life Science and Natural Resources, Korea University, Seoul, South Korea; ^2^Department of Plant Biotechnology, Graduate School, Korea University, Seoul, South Korea; ^3^Smart Farm Research Center, Korea Institute of Science and Technology, Gangneung Institute, Gangneung, South Korea; ^4^Division of Biotechnology, College of Life Sciences and Biotechnology, Korea University, Seoul, South Korea

**Keywords:** anti-virulence, immune-priming, type III secretion system (TT3S), resveratrol oligomers, stilbene

## Abstract

Antibiotic resistance has become increasingly prevalent in the environment. Many alternative strategies have been proposed for the treatment and prevention of diverse diseases in agriculture. Among them, the modulation of bacterial virulence to bypass antibiotic resistance or boost plant innate immunity can be considered a promising drug target. Plant-produced natural products offer a broad spectrum of stereochemistry and a wide range of pharmacophores, providing a great diversity of biological activities. Here, we present a perspective on the putative role of plant-produced resveratrol oligomers as anti-virulence and plant-immune priming agents for efficient disease management. Resveratrol oligomers can decrease (1) bacterial motility directly and (2) indirectly by attenuating the bacterial type III secretion system (TT3S). They induce enhanced local immune responses mediated by two-layered plant innate immunity, demonstrating (3) a putative plant immune priming role.

## Introduction

Resveratrol is a plant polyphenol stilbene distributed widely in plant families including Dipterocarpaceae, Vitaceae, Cyperaceae, Fabaceae, Gnetaceae, and Paeoniaceae (He and Yan, 2013; Pecyna et al., 2020). Resveratrol is a bioactive phytoalexin whose levels increase in response to biotic and abiotic stresses such as fungal infection, exposure to ultraviolet (UV) light, and wounding (Jeandet et al., 1995). The concentration of resveratrol in plants can thus be a useful indicator of disease resistance, as it constitutes both a constitutive and an inducible defense mechanism (Niesen et al., 2013).

Resveratrol has been extensively studied for several of its biological properties that are beneficial to human health, such as its antioxidant, anti-inflammatory, and cancer chemo-preventive activities (Gülçin, 2010). Further studies have focused on stilbene derivatives and their potential role in both plant and human health (El Khawand et al., 2018). Stilbene compounds exhibit promising antibacterial activity against several Gram-positive bacteria, including methicillin-resistant *Staphylococcus aureus* (MRSA) and vancomycin-resistant *Enterococcus faecalis* (VRE) (Man et al., 2018; Goddard et al., 2020). For the plant disease management, extracts from *Rheum rhabarbarum* are rich in three stilbenes (rhaponticin, desoxyrhaponticin, and resveratrol) that block mycelial growth of plant-pathogenic fungi and oomycetes *in vitro* (Gillmeister et al., 2019).

Stilbene derivatives undergo structural modifications, such as polymerization, oxidation, glycosylation, and substituent rearrangement in plants leading to extensive structural diversity and various degrees of bioactivity (Niesen et al., 2013). Resveratrol oligomers consisting of two to eight resveratrol monomers are one of the largest groups of stilbenes, and more than 29 different oxidative coupling processes are required to generate diverse skeletons and complex configurations of resveratrol oligomers from monomers (Cichewicz and Kouzi, 2002; He and Yan, 2013). Resveratrol oligomers are synthesized as biological defense compounds in plants and accumulate at the site of lesion or infection (Keylor et al., 2015). Although resveratrol monomers do not have antifungal activity, resveratrol oligomers possess potent antifungal properties and accumulate more in grapevine resistant to the Botryosphaeriaceae family of fungi (Langcake, 1981; Lambert et al., 2012). Resveratrol oligomers can regulate the transcriptional level of type III secretion system (T3SS) genes of the Gram-negative bacterium *Pseudomonas syringae* pv. *tomato* DC3000 (*Pst* DC3000) (Kang et al., 2020). In addition, pterostilbene was the most active antifungal compound against the bacterium *S. aureus in vitro* among nine tested resveratrol analogs (Zakova et al., 2018). In this perspective, we briefly review and explore the putative roles of resveratrol compounds as anti-virulence and plant-immune priming agents for broad-spectrum plant disease control agents.

### Modulation of Bacterial Virulence by Stilbene Compounds

Antibiotic-resistant pathogens require alternative strategies for their control and mitigation. An anti-virulence approach has been proposed as a promising alternative strategy to block an infection by a bacterial pathogen by neutralizing virulence factors, including toxin production, biofilm formation, quorum sensing (QS), two-component systems (TCSs), and the T3SS (Dickey et al., 2017). In addition, plant-derived compounds with anti-virulence activities against pathogenic bacteria have been reported as candidates for new drugs with potential medical applications (Joshi et al., 2021). In this section, we highlight the utilization of plant-derived stilbene compounds with anti-virulence activities against pathogenic bacteria.

Bacteria use QS as a cell-to-cell communication system to maintain population size using an extracellular signal molecule (*N-*acyl-homoserine lactone, AHL) as cell density increases (Joshi et al., 2021). The QS system allows the pathogens to activate the expression of virulence genes responsible for biofilm formation, extracellular enzymes, and motility. Plant phenolic compounds constitute the largest group of QS inhibitors (Hossain et al., 2017; Muñoz-Cazares et al., 2017), including salicylic acid and cinnamic acid. These two phenolic compounds decrease AHL levels *in vitro* in pathogenic bacteria including *Pseudomonas aeruginosa*, *S. aureus*, and *Pectobacterium* spp. (Rajkumari et al., 2018; Zhang et al., 2018; Joshi et al., 2020; Dotto et al., 2021). These two phenolics also affect the transcriptional pattern of QS genes and QS-related genes in *P. aroidearum* PC1 and *P. carotovorum* spp. *brasiliense* Pcb 1692 by interfering with the accumulation of AHL (Joshi et al., 2016).

Some stilbenes, which are a major group of polyphenols, modulate QS in various bacterial pathogens. Of those stilbenes, resveratrol, piceatannol, and oxyresveratrol diminish the biosynthesis of AHL in *Chromobacterium violaceum* CV026 and lower QS-controlled toxin production and swarming motility in *P. aeruginosa* PAO1 *in vitro* (Sheng et al., 2015). The resveratrol dimer ε-viniferin and *trans-*resveratrol isolated from *Carex pumila* inhibit biofilm formation in *P. aeruginosa* PA14 (Cho et al., 2013). Qin et al. (2014) suggested that inhibition of biofilm formation by resveratrol may be accomplished *via* disruption of QS and biosynthesis of surface proteins and capsular polysaccharides, based on a transcriptome analysis in *S. aureus.*

**FIGURE 1:**
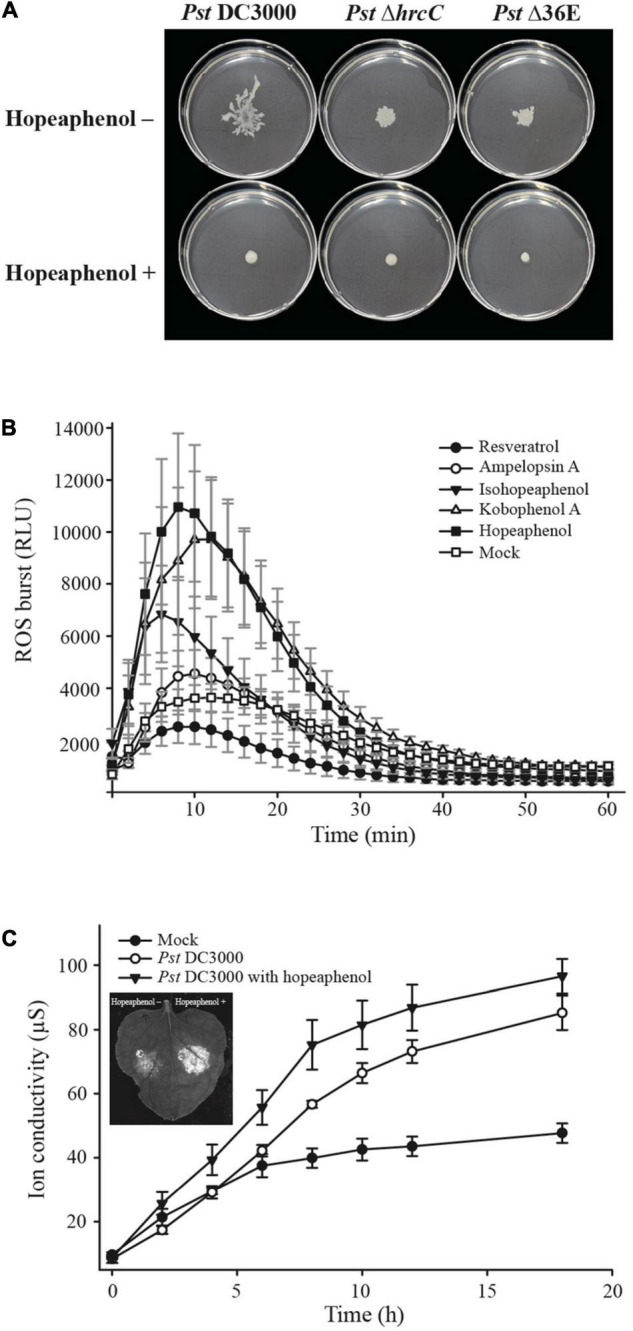
Resveratrol compounds regulate bacterial motility, ROS production, and induction of cell death response. **(A)** Bacterial motility upon hopeaphenol treatment. The *Pst* DC3000 wild-type strain, the *hrcC* deletion mutant (*Pst* Δ*hrcC*), and a mutant lacking 36 effector genes (*Pst*Δ36E) fail to swarm on the surface of a medium containing hopeaphenol. Tendril patterns and migrating distance were observed 48 h after inoculation. **(B)** ROS burst in Arabidopsis in response to pre-treatment with resveratrol compounds. Arabidopsis leaves were pre-treated with five different resveratrol derivatives at a concentration of 100 μM, 19 h before exposure to PAMP. ROS production was measured with a luminometer every 2 min for 1 h. **(C)** Cell death induction in *N. benthamiana* leaves. Hopeaphenol was applied to *N. benthamiana* leaves 16 h before infection. The ion conductivity of the leaves infected by *Pst* DC3000 (1 × 10^6^ cfu/ml) was measured 40 h after infection. The picture on the left of the graph showed induced cell death in *N. benthamiana* leaf upon pre-treatment with hopeaphenol.

The bacterial secretion system is another major anti-virulence target for disease management. The T3SS is required for the delivery of type III effectors (T3Es) mainly in Gram-negative bacteria (Lindeberg et al., 2006). T3Es are translocated through the T3SS, a syringe needle–like apparatus encoded in the pathogenicity island (Alfano et al., 2000). Once translocated into host cells, T3Es modulate the defense system to support successful pathogen colonization. Hence, recent research focusing on T3SS inhibitors has provided an impetus for the development of agents for anti-virulence. The resveratrol tetramer hopeaphenol is one such well-characterized plant-produced compound among stilbenes with T3SS inhibitory properties. Hopeaphenol was first identified as a T3SS inhibitor from two Papua New Guinean rainforest plants, *Anisoptera thurifera* and *A. polyandra* (Zetterström et al., 2013). Treatment with hopeaphenol inhibited the secretion of the effector protein YopE in *Yersinia pseudotuberculosis in vitro* without causing growth retardation and lowered the expression of ExoS, encoding a protein with a YopE-like GAP (GTPase activating protein) domain and with similar cytotoxic activity in *P. aeruginosa* (Zetterström et al., 2013). Hopeaphenol was identified as being highly effective in repressing the promoter activity of the T3SS pilus gene *hrpA* from the plant pathogenic bacterium *Pst* DC3000, while hopeaphenol, isohopeaphenol, and ampelopsin A (a resveratrol dimer) downregulated the expression of several T3SS-related genes *in vitro* and decreased disease severity *in vivo* in tomato (*Solanum lycopersicum*) plants (Kang et al., 2020). Hopeaphenol also decreased the transcription of two genes encoding AraC-type transcriptional activators of T3SS gene and T3SS pilus gene expression in *Ralstonia solanacearum in vitro*, the causal agent of bacterial wilt disease (Puigvert et al., 2019). Thus, hopeaphenol and other stilbenes may be attractive potential T3SS inhibitors for the control of bacterial diseases. Sundin et al. (2020) utilized a green fluorescent protein (GFP)-expressing pathogen to screen hopeaphenol-related chemical compounds, including resveratrol dimers and stilbenoid natural products and analogs, to discover virulence-blocking agents. In particular, hopeaphenol analogs demonstrated moderate inhibitory activities *in vitro* on ExoS expression in *P. aeruginosa* and on effector secretion in *Y. pseudotuberculosis* (Sundin et al., 2020). Likewise, *Pst* DC3000 expressing GFP under the control of the *hrpA* (T3SS pilus gene) promoter with the treatment of stilbenes, including resveratrol dimer, tetramer, glycoside, and resveratrol derivatives condensed with a flavanone, displayed reduced GFP intensity, indicating potential T3SS inhibitory activity by these compounds (Kang et al., 2020). Notably, treatment with hopeaphenol and kobophenol A downregulated the expression of *hrpA*, *hrpL*, and *hopP1* genes in the *hrp* cluster *in vitro* (Kang et al., 2020). We also determined that hopeaphenol limits *Pst* DC3000 bacterial motility *in vitro* (Figure 1A). An intriguing result was lower bacterial motility without hopeaphenol treatment in the *hrcC* deletion mutant defective in the T3E delivery machine and *Pst* DC3000 D36E mutant lacking 36 T3E genes compared with the motility in wild-type (*Pst* DC3000), which is a phenocopy of hopeaphenol treatment on wild-type *Pst* DC3000. Thus, we highlight that the repression of the expression of T3SS or T3Es gene as novel anti-virulence targets (Figure 1A) and should be investigated further.

Together, the application of bioactive stilbene compounds such as resveratrol and derivatives may accomplish appropriate levels of bacterial disease control and therefore is a promising strategy to bypass antibiotic resistance.

### Plant Immune Priming-Natural Products

Plants defend themselves against pathogen attacks through a two-layered innate immunity (Jones and Dangl, 2006). The first layer of plant immunity relies on recognizing conserved pathogen molecules, so-called pathogen-associated molecular patterns (PAMPs) *via* cell surface–localized immune receptors known as pattern recognition receptors: this is PAMP-triggered immunity (PTI). The second layer of the plant innate immune response is known as effector-triggered immunity (ETI) and is controlled by nucleotide-binding leucine rich-repeat (NLR) immune receptors that recognize, either directly or indirectly, effector proteins secreted by pathogens (Eitas and Dangl, 2010; Jones et al., 2016). These two plant immune responses play an essential role in plant tissues infected by pathogens locally to initiate systemic signaling that reaches uninfected plant leaves and tissues, leading to systemic resistance (Fu and Dong, 2013). In addition, plant root–colonizing non-pathogenic bacteria can induce a long-distance signal that initiates systemic resistance in plants (Zehra et al., 2021). The resulting induced resistance is classified as systemic-acquired resistance (SAR) or induced systemic resistance (ISR) based on the cause of the initial activation: local foliar pathogen infection (SAR) or beneficial bacteria in the plant rhizosphere (ISR) (Van der Ent et al., 2009; Fu and Dong, 2013). Priming is a response to signals from microbes, plants, or pathogen effectors, and natural or synthetic compounds (Mauch-Mani et al., 2017). Priming improves the immune capacity to initiate plant immune responses in systemic tissues against subsequent biotic or abiotic stress (Conrath, 2011). Thus, priming represents a general induced resistance phenomenon in plants that encompasses both SAR and ISR.

In this section, we focus mainly on plant immune priming by chemical compounds, as they produce good reproducibility in terms of induced resistance. Salicylic acid (SA) is a plant hormone that modulates local and systemic immune response in plants (van Hulten et al., 2006; Wilson et al., 2014; Gully et al., 2019). Likewise, another priming inducer, benzo-(1,2,3)-thiadiazole-7-carbothioic acid *S*-methyl ester (BTH), mediates the activation of callose deposition and expression of genes encoding phenylalanine ammonia-lyase (PAL) responsible for the biosynthesis of defense-related secondary metabolites such as phytoalexins and lignin-like polymers, leading to broad-range resistance against pathogens (Hahlbrock and Scheel, 1989). Natural secondary metabolites also mediate induced resistance in plants, including jasmonic acid (JA), methyl salicylate, pipecolic acid, dehydroabietinal, and glycerol-3-phosphate (Holmes et al., 2019). Recent studies have focused on identifying plant resistance-inducing compounds through extensive high-throughput screening of chemical libraries derived from synthetic or natural sources (Aranega-Bou et al., 2014; Zhou and Wang, 2018).

Pathogen-associated molecular patterns (PAMPs) such as bacterial peptidoglycans, flagellin, chitin, polysaccharides, and other membrane or cell wall components from various pathogens serve as elicitors of local plant innate immune responses, subsequently boosting induced resistance in plants (Livaja et al., 2008; Zehra et al., 2021). Laminarin is a polysaccharide isolated from the brown algae *Laminaria digitata* that is perceived by several plants (Inui et al., 1997; Cardinale et al., 2000). Laminarin stimulates an extensive array of early- and late-defense reactions in tobacco and grapevine cells such as extracellular alkalinization, activation of the phenylpropanoid pathway, and SA accumulation (Klarzynski et al., 2000; Aziz et al., 2003). Consistent with its role as a plant immune elicitor, laminarin induces the accumulation of pathogenesis-related (PR) proteins in tobacco and grapevine leaves, enhancing plant disease resistance against phytopathogens (Klarzynski et al., 2000; Aziz et al., 2003). Similarly, many secondary metabolites derived from natural sources have plant immune priming activity. For instance, hexanoic acid, a natural flavor component of strawberry, has immunity priming, and antimicrobial properties against plant pathogens such as *Botrytis cinerea* and *Pst* DC3000 in tomato (Zabetakis et al., 2000; Leyva et al., 2008; Scalschi et al., 2013). Hexanoic acid counteracts the action of the phytotoxin coronatine, which functions as a jasmonyl-isoleucine mimic and suppresses SA-dependent defenses (Uppalapati et al., 2007; Scalschi et al., 2013). Root extracts from *R. rhabarbarum* induce priming in Arabidopsis by activating the local and systemic expression of *PR* genes (Gillmeister et al., 2019). Another stilbene-rich extract obtained from grape canes also induces immune responses by activating mitogen-activated protein kinase (MAPK) and defense-related gene expression such as *PR* and *Glutathione-S-transferase 1* (*GST1*) genes (De Bona et al., 2019). To test whether resveratrol derivatives can enhance more plant innate immune responses, we examined flg22-triggering reactive oxygen species (ROSs) burst in *Arabidopsis thaliana* and *Pst* DC3000-inducing cell death phenotype in *Nicotiana benthamiana*, demonstrating key immune responses of two-layered plant immunity. Among five tested resveratrol derivatives, kobophenol A and hopeaphenol (resveratrol tetramers) substantially enhanced more flg22-mediated ROS burst in Arabidopsis, a hallmark of early PTI responses (Figure 1B). Moreover, treatment with hopeaphenol (one of the resveratrol tetramers) increased *Pst* DC3000-induced cell death responses in *Nicotiana benthamiana* compared with non-treated controls, leading us to infer that TIR-NB-LRR Roq1-mediated immune response by recognizing *Pst* DC3000 type III effector *hopQ1-1* may be enhanced by hopeaphenol treatment (Schultink et al., 2017; Thomas et al., 2020; Figure 1C). Our observations of enhanced ROS burst triggered by flg22 and faster cell death response upon *Pst* DC3000 infection on *Nicotiana benthamiana* with resveratrol oligomers suggest that some stilbene compounds can enhance plant immunity (Figures 1B,C). We, therefore, propose the natural compounds, resveratrol oligomers may potentiate plant-immune priming events by enhancing stronger local immune responses and consider these compounds as putative plant disease control agents for sustainable agriculture.

## Discussion

Despite common and distinct innate immune activation in plants *via* PTI and ETI, these two immune systems are considered to have evolved during the arms race between pathogens and their host plants, as reflected by the two different classes of immune receptors involved and distinct initiation of early immune signaling (Jones and Dangl, 2006; Nishimura and Dangl, 2010). Recent studies suggest that these two layers of plant innate immunity converge and work coordinately to evoke robust immune responses, as ETI demonstrates a stronger immunity upon PTI initiation (Tena, 2021; Yuan et al., 2021; Ngou et al., 2022). This observation raises the possibility that robust PTI responses at the local site of pathogen infection can have a synergistic effect on ETI activation and produce stronger long-distance signals to boost systemic resistance against further pathogen infections. We thus hypothesize that, upon new pathogen infection in plant tissues systemically activated by a prior pathogen attack, greater immune priming in combination with stronger local PTI and ETI activation at the newly infected site can provide an efficient strategy for the sustainable control of plant diseases.

**FIGURE 2:**
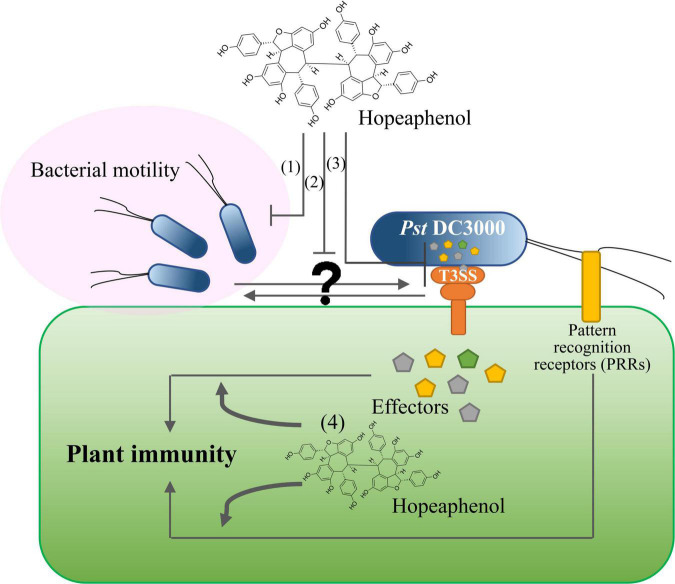
Hopeaphenol has a dual role in bacterial virulence and plant innate immunity. (1) Hopeaphenol suppresses the virulence activity of pathogens by directly inhibiting bacterial motility. (2, 3) Hopeaphenol modulates the expression of genes encoding effector proteins, subsequently alleviating pathogen virulence. (4) Hopeaphenol may enhance local immune responses mediated by two-layered plant innate immunity.

The identification of natural compounds as biological control agents against plant diseases has been largely limited to those exhibiting anti-microbial activity to eradicate pathogens or to those enhancing plant resistance by restricting pathogen growth at infection sites (Singh and Yadav, 2020). However, the use of bactericidal treatment leads to the emergence of chemical-resistant pathogens (Silva et al., 2016). The resveratrol tested here demonstrated successful repression of bacterial type III–dependent gene expression and bacterial motility (Figure 1A) and no anti-microbial activity (Kang et al., 2020). The capacity of resveratrol as a biological control agent is thus solely because of its suppression of pathogen virulence, which may be less prone to the emergence of new resistant strains upon exposure to this compound. We also determined that T3SS is likely to be associated with bacterial motility, as *Pst* DC3000 mutants lacking T3SS (*hrcC* or D36E) failed to swarm along the medium surface, in contrast to the wild-type strain. This result strongly suggests that resveratrol and perhaps its derivatives can be utilized as efficient biological control agents with dual functions, as illustrated in Figure 2. Hopeaphenol may suppress pathogen virulence *via* direct inhibition of bacterial motility (1) or by modulating the expression of genes encoding bacterial effectors, subsequently alleviating pathogen virulence along with lower motility (2 and 3) and enhancing local immune responses mediated by the orchestration of the two layers of the plant innate immunity (4). We anticipate that a comprehensive examination of the output of PTI and ETI responses upon plant treatment with hopeaphenol, along with priming-dependent systemic resistance, will provide intriguing evidence in the future. An intensive investigation of how T3SS and effector gene expression can influence bacterial motility will be necessary, considering our swarming behavior results (Figure 1). Natural compounds such as biological control agents may thus offer new research directions to realize broad-spectrum protection against pathogens in agriculture.

## Data Availability Statement

The original contributions presented in the study are included in the article/Supplementary Material, further inquiries can be directed to the corresponding author/s.

## Author Contributions

JK and NY performed the experiments. JK, NY, BJ, and E-HC analyzed the results. JK and E-HC conceptualized and wrote the draft. JK, NY, BJ, BK, and E-HC edited the final manuscript. All authors contributed to the article and approved the submitted version.

## Conflict of Interest

The authors declare that the research was conducted in the absence of any commercial or financial relationships that could be construed as a potential conflict of interest.

## Publisher’s Note

All claims expressed in this article are solely those of the authors and do not necessarily represent those of their affiliated organizations, or those of the publisher, the editors and the reviewers. Any product that may be evaluated in this article, or claim that may be made by its manufacturer, is not guaranteed or endorsed by the publisher.
