# Supplementary material for: Resveratrol Oligomers, Plant-Produced Natural Products With Anti-virulence and Plant Immune-Priming Roles
Source: Front Plant Sci. 2022 May 27;13:885625. doi: 10.3389/fpls.2022.885625 (PMC9197177; doi:10.3389/fpls.2022.885625)
Supplement: Supplementary file 1 [file Data_Sheet_1.pdf]

Supplementary methods

Bacterial motility

Overnight cultures of *Pst* DC3000, *Pst*Δ*hrcC* and *Pst* D36E in King'B medium containing 20 g of proteose peptone no.3, 1.5 g of K_2HPO_4, 1.5 g of $MgSO_4 \cdot 7H_2O$, 15 mL of glycerol and 15 g of agar per liter were adjusted 2.0 of optical density at 600 nm. The bacterial suspensions were inoculated onto a motility medium containing 0.5 g of proteose peptone no.3, 0.3 g of yeast extract, and 0.4 g of agar per liter. Hopeaphenol was supplemented into the medium with 100 μM. 48 hours after incubation at 28 °C, the distance of bacterial movement and morphology of bacterial tendrils were observed.

ROS burst measurement

Resveratrol derivatives (resveratrol, ampelosin A, isohopeaphenol, kobophenol A, and hopeaphenol) and sterilized water (mock) were infiltrated into four-weeks-old Arabidopsis leaves at the concentration of 100 μM. 16 hours after infiltration, the leaf disc was cut using no.2 cork borer and was immersed in 96-well white plate containing 200 μL of sterilized water, followed by incubation at room temperature for 3 hours. The water was carefully discarded, and 200 μL of reaction solution containing 100 nM of PAMP (*P. aeruginosa* flg22), 0.2 μM of luminol, and 20 μg mL^{-1} of horseradish peroxidase was quickly added to each with leaf disc. ROS production was evaluated by Luminometer (Centro, Berthold, Germany) every 2 min for 1h.

Cell death measurement

Six-week-old *N. benthamiana* plants were pre-treated with sterilized water or 100 μM of hopeaphenol by syringe infiltration. After 16 h, bacterial suspension of *Pst* DC3000 (1×10^6 CFU mL^{-1}) was infiltrated at the same site treated with water or hopeaphenol. The leaf disc was cut by no.3 cork borer, and 4 leaf discs per each treatment were put into a round bottom plastic tube containing 6 mL of sterilized water 40 hours after infection. The ion conductivity was measured every 2 h by a conductivity meter (VWR, USA). The photo of the representative result was taken 44 hours post-infection.
